# Cutaneous leishmaniasis: A case report of a diagnostic dilemma

**DOI:** 10.1002/ccr3.5428

**Published:** 2022-02-13

**Authors:** Prajwal Pudasaini, Prashanta Pudasaini

**Affiliations:** ^1^ Department of Dermatology Gandaki Medical College Teaching Hospital (GMCTH) Pokhara Nepal; ^2^ Department of General Surgery Kathmandu Medical College Teaching Hospital (KMCTH) Kathmandu Nepal

**Keywords:** cutaneous leishmaniasis, fine‐needle aspiration cytology

## Abstract

Cutaneous leishmaniasis (CL) is the most prevalent clinical form of leishmaniasis and is caused by vector‐borne protozoan parasite. Variation in diagnostic accuracy exists. A 54‐year‐old female farmer by occupation presented with lesion over right thigh for 8 months. Lesion evolved over period of 2–3 months and progressed to form ulcer with surrounding redness. On examination, solitary plaque with crateriform ulcer 3 * 2 cm in size roughly oval in shape was present. Ulcer floor was moist, smooth shiny with serous discharge, and well‐defined raised erythematous margin was present. Biopsy was done which showed features suggestive of lupus vulgaris, for which anti‐tubercular treatment (ATT) was started. There was persistence of ulcer despite 4 months of ATT, for which diagnosis was reconsidered and fine‐needle aspiration cytology (FNAC) was performed. FNAC showed numerous intra‐ and extracellular amastigotes suggestive of leishmaniasis which was treated with complete disappearance of ulcer over 4 months.

## Graphical abstract

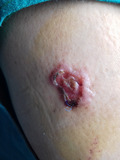

## 1 BACKGROUND

Cutaneous leishmaniasis (CL) is the most prevalent clinical form of leishmaniasis and is caused by vector‐borne protozoan parasite.^1^ Variation in diagnostic accuracy exists between different parasitological and histopathological examination.^2^ Test of choice for diagnosis depends not only on the sensitivity and specificity of a test but also on the availability, especially in a resource‐poor setting. CL is difficult for the clinicians to diagnose because of the rarity of the disease and non‐specific presentation. Here, we report a rare case report of CL.

## 2 OBSERVATION

**FIGURE 1:**
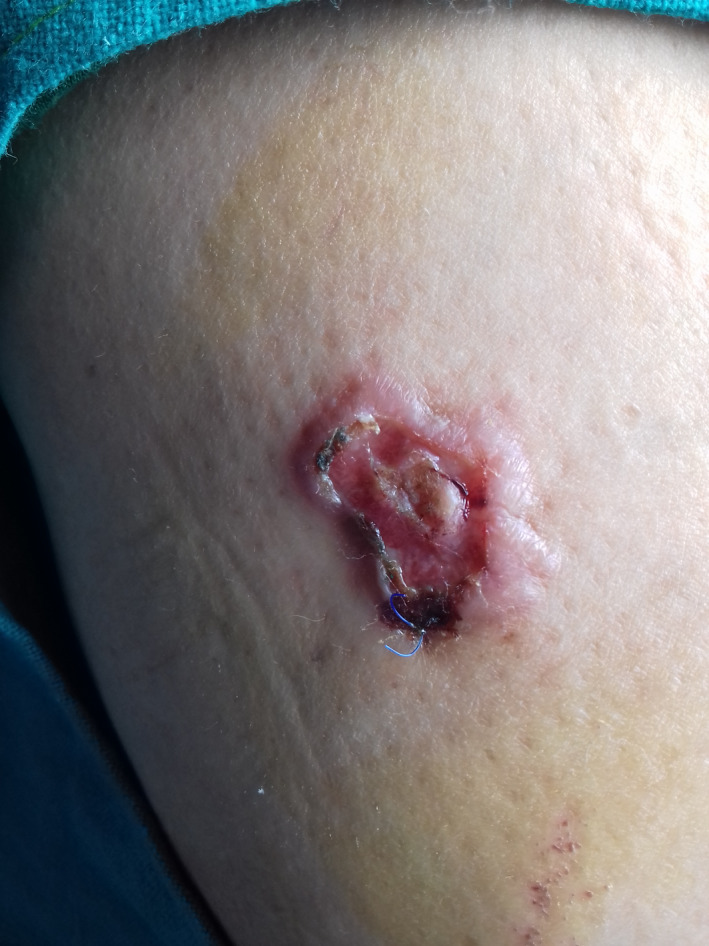
Solitary crateriform ulcer with erythematous halo of cutaneous leishmaniasis over right thigh, upper third aspect in the lateral part

A 54‐year‐old female patient from Gorkha, farmer by occupation with frequent outdoor activities, presented with lesion over right thigh for 8 months. Initially, single pinhead sized, soft, raised lesion with brownish red color was noted over right thigh that increased in size with crust formation. Lesion evolved over period of 2–3 months and progressed to form ulcer with surrounding redness. On examination, solitary plaque with crateriform ulcer 3 * 2 cm in size roughly oval in shape was present over the right thigh, upper third aspect in the lateral part, approx. 15 cm from Anterior Superior Iliac Spine (ASIS). Ulcer floor was moist, smooth shiny with serous discharge, and well‐defined raised erythematous margin was present (Figure 1). Biopsy was done which showed features suggestive of lupus vulgaris, for which anti‐tubercular treatment (ATT) was started. There was persistence of ulcer despite 4 months of ATT, for which diagnosis was reconsidered and fine‐needle aspiration cytology (FNAC) was performed. FNAC showed numerous intra‐ and extracellular amastigotes suggestive of leishmaniasis, which was treated with complete disappearance of ulcer over 4 months.

## 3 DISCUSSION

As CL is rare and given the limitation of available diagnostic modalities in a resource‐poor setting, diagnosis can be confusing. Diagnosis can be made with good clinical acumen and FNAC in an endemic area.^3^ With proper diagnosis, overall prevalence of the disease can be estimated and clinical therapeutic trials can be performed with timely prevention of mucocutaneous complications.

## CONFLICT OF INTEREST

None.

## AUTHOR CONTRIBUTIONS

Section author assisted the primary author during biopsy, and in the process of case report writing and discussion.

## CONSENT

Hereby, I Dr Prajwal Pudasaini, MD consciously assure that for the manuscript CUTANEOUS LEISHMANIASIS: A CASE REPORT OF A DIAGNOSTIC DILEMMA author has confirmed during submission that patient consent has been signed and collected in accordance with the journal’s patient consent policy.

## Data Availability

The data that support the findings of this study are openly available in clinical case reports at https://doi.org/10.22541/au.163252740.02365320/v1.
